# Comparison of different waste bin monitoring approaches: An exploratory study

**DOI:** 10.1177/0734242X231160691

**Published:** 2023-05-03

**Authors:** Yoeri Brouwer, Ana Paula Barbosa-Póvoa, António Pais Antunes, Tânia Rodrigues Pereira Ramos

**Affiliations:** 1 Department of Electrical and Computer Engineering, Instituto Superior Técnico, Universidade de Lisboa, Lisbon, Portugal; 2 Centre for Management Studies (CEGIST), Instituto Superior Técnico, Universidade de Lisboa, Lisbon, Portugal; 3 CITTA, Department of Civil Engineering, University of Coimbra, Coimbra, Portugal

**Keywords:** Smart cities, monitoring solutions, waste management, collection efficiency, service level

## Abstract

Waste bin monitoring solutions are an essential step towards smart cities. This study presents an exploratory analysis of two waste bin monitoring approaches: (1) ultrasonic sensors installed in the bins and (2) visual observations (VO) of the waste collection truck drivers. Bin fill level data was collected from a Portuguese waste management company. A comparative statistical analysis of the two datasets (VO and sensor observations) was performed and a predictive model based on Gaussian processes was applied to enable a trade-off analysis of the number of collections versus the number of overflows for each monitoring approach. The results demonstrate that the VO are valuable and reveal that significant improvements can be achieved for either of the monitoring approaches in relation to the current situation. A monitoring approach based on VO combined with a predictive model is shown to be viable and leads to a considerable reduction in the number of collections and overflows. This approach can enable waste collection companies to improve their collection operations with minimal investment costs during their transition to fully sensorized bins.

## Introduction

Waste management is a serious challenge wherever there are large populations. Furthermore, the global trend is for people to move into urban areas (Pereira and Navarro, 2015; Weeks, 2007). This trend, coupled with rising global development levels, increases the amount of waste generated in cities and towns (Hannan et al., 2015). Therefore, there is a growing need for municipalities to increase the efficiency of their waste management systems.

In recent years, several integrated solutions have been developed for waste management systems, including bin sensors, servers for gathering and analysing sensor information and applications to collect information about bin states and even to plan collection routes (Gutierrez et al., 2015; Karadimas et al., 2016; Mamun et al., 2016; Medvedev et al., 2015; Murciego et al., 2015; Ramson and Moni, 2017; Rovetta et al., 2009; Shah et al., 2018; Yerraboina et al., 2018). Having access to real-time information about bin fill levels is a valuable feature that can be performed accurately and automatically without the need for human intervention. It allows waste collection companies to save operational costs by reducing the number of trips that need to be made, as well as by reducing the total length of those trips (Morais et al., 2023). Automatic monitoring systems can also issue a warning when a bin is close to overflowing or when it has overflowed, therefore increasing the quality of service for all citizens.

Currently, most municipalities are not utilizing smart waste management systems. Instead, they usually rely on pre-planned routes travelled on regular intervals, which are often designed manually, regardless of the quantities of waste to collect (Ferrer and Alba, 2019). This leads to routes which are far from optimal and do not adjust to changes in waste quantities or travel conditions (e.g. traffic congestion). Moreover, route optimization for waste collection is a challenging problem, as waste quantities are stochastic (Prins et al., 2014) and, without a monitoring solution (such as sensorization), they are only known a posteriori. Studies have shown that waste generation and recycling rates are dependent on socio-economic, demographic and geographic factors (Abbasi and El Hanandeh, 2016; Hannan et al., 2015; Kannangara et al., 2018; Prins et al., 2014). In fact, as regions become more developed and consumer expenditure increases, so too does the amount of generated waste (Kannangara et al., 2018). Therefore, urban growth and national development must be accompanied by upgrades in municipal waste management systems, especially with regards to waste collection.

To address inefficiency issues, two approaches can be employed. One is the installation of smart sensors in bins, coupled with route optimization techniques (Gutierrez et al., 2015; Medvedev et al., 2015; Murciego et al., 2015; Ramos et al., 2018; Shah et al., 2018). However, sensors are not cheap (the market price of commercially available sensors can be one to two orders of magnitude (Beigl et al., 2005; Vishnu et al., 2022) and can require a considerable investment from municipalities, which are often responsible for hundreds if not thousands of bins. The other approach relies on robust and adaptive models for predicting fill levels (Ferrer and Alba, 2019). This approach depends on historic data for individual bin fill levels, which can be difficult to gather. Some waste management companies have their drivers recording this data every time a bin is visited. However, as the fill levels are registered by humans, and in a discrete scale, it may have lower precision and may be prone to individual subjectivity and human error. Moreover, if fill levels are only recorded when drivers visit the bins, then the time interval between observations can be considerable, especially for recyclable waste. The pros and cons of these two approaches (installation of smart sensors in bins – static sensors (SS); predicting fill levels based on visual observations (VO) from the truck drivers – VO) are systematized in Table 1.

**Table 1.** Comparison of monitoring approaches.

Monitoring approach	Pros	Cons
Static sensors	A priori fill level knowledge (real-time information) Accurate and precise fill level measurements	Large initial investment Considerable maintenance and licensing costs
Visual observations	Negligible cost	A posteriori information Requires a short-term predictive model Susceptible to human error Low measurement precision

In this work, we perform an exploratory study where we analyse monitoring approaches by installing smart sensors in recyclable bins in a living laboratory environment, while simultaneously asking collection truck drivers to record the fill levels of the respective bins. Our goal is to compare their performance, that is, to compare visual fill level observations made by humans with those made by sensors and propose a hybrid monitoring approach that balances the advantages and disadvantages of the other two approaches. Then, the impact of each monitoring approach is assessed (considering that the data is provided by sensors or by humans) when planning the waste collection operations, namely in terms of the number of collections and the number of overflows. These two figures are inevitably tied together, as a reduction in one usually leads to an increase in the other. Furthermore, the number of collections made is strongly related to the efficiency of waste management operations, whereas the number of overflows directly impacts the company’s service level (more overflows imply a reduced service level).

The contributions of our study are as follows:

1. Analysis of two monitoring approaches of bin fill levels: Visual monitoring by waste collection truck drivers and SS monitoring. This includes a comparative analysis of sensor and VO, based on which the quality of the VO is assessed (quantitatively);
2. Proposal and analysis of a novel monitoring approach where truck drivers use a mobile sensor (MS) to measure bin fill levels;
3. Comparison of the three monitoring approaches via an analysis of the trade-off between the number of collections made and the number of overflows that occur, using real data from a living laboratory and
4. Managerial insights for waste collection companies regarding the pros and cons of each monitoring approach, and their expected impact in terms of reduction in the number of collections (efficiency) and reduction in the number of overflows (service level).

It should be noticed that our study is exploratory, and its results are preliminary. However, a second testing phase at a wider scale (both temporal and spatial, including more representative data – more waste bins located at different regions, all seasons, more truck drivers involved) is foreseen in order to retrieve more general results.

The remainder of the paper is organized as follows: section ‘Study methodology’ describes the methodology we have used in our study; in section ‘Study results’, the results of the analyses we have performed are presented and discussed in detail; finally, section ‘conclusion’ states our main conclusions.

## Study methodology

**Figure 1.**
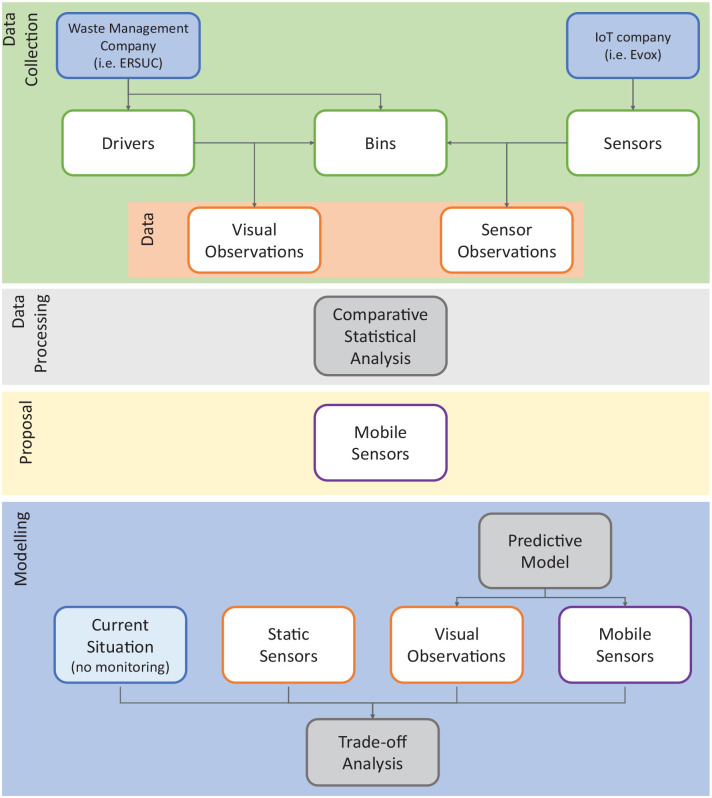
Major steps of the study methodology.

The methodology used to assess the monitoring approaches comprises four major steps: data collection, data processing, proposal, and modelling (see Figure 1).

### Data collection

Regarding data collection, bin fill level data was collected over the course of 7 months in a living laboratory scenario from two sources: observations made by fill level sensors installed in the bins (sensor observations) and observations made by the waste collection truck drivers (VO). The monitored bins are owned by ERSUC – Resíduos Sólidos do Centro, S.A., a Portuguese waste management company operating in 36 municipalities of the Coimbra and Aveiro regions (ERSUC, 2018). The installed sensors were provided by Evox, a Portuguese company providing technological IoT solutions for several industries (Evox Technologies, 2019). The sensors used in this experiment were ultrasonic sensors installed inside the bins at the top, pointing downward. The sensors have a resolution of ±1 cm and a ranging distance between 18 and 400 cm. The cone formed by the ultrasonic beam has an aperture of 80°. Each sensor operates on a lithium battery and communicates with a central server via Long Term Evolution network.

In our exploratory study, four different recyclable waste bins were monitored from 15 February 2019 to 30 September 2019: two paper/cardboard bins (hereafter referred to as paper 1 and paper 2), one plastic/metal bin (plastic) and one glass bin (glass). The bins are all located in the same street, where the glass, plastic and paper 1 bins are grouped at the same location, while paper 2 bin is at another location on the same street. The four bins have the same geometry, a total capacity of 2.5 m^3^ and the dimensions illustrated in Figure A-1 (Supplemental Appendix). These bins were selected due to their central position in the city of Coimbra (150,000 inhabitants), meaning that they could be regularly monitored by the truck drivers. As reported by ERSUC, they are characterized by a high and stable fill rate over the year. Although the whole area of ERSUC (36 municipalities) is somehow affected by seasonality (in particular glass, see Figure A-2, Supplemental Appendix), this is not the case of the selected bins given their location and urban environment.

Two different methods were used to collect data on bin fill levels. Firstly, fill level sensors were installed in each of the four bins. These sensors autonomously monitor changes in the fill level and transmit collected data to a remote server. The most important data for the purposes of our study were the bin identifier, the fill level and the time and date. Figure A-3 (Supplemental Appendix) illustrates the installation process of one of the sensors. The recorded sensor data was subsequently downloaded from the remote server for visualization and analysis. It should be noted that the sensors are continuously reading the fill levels, but only transmit a level when it is significantly different from the level measured in previous transmission for battery saving purposes. A significant change was parametrized in this experiment to be ±10 percentage points, that is, if the previous transmission at 3 p.m. was 35%, and at 4 p.m. the level is 40%, at 4 p.m. there will be no transmission. If, instead, at 4.30 p.m. the level is 45%, then the fill level will be transmitted. If no significant changes occur, the sensors report fill levels at least once a day.

In parallel with the sensor monitoring system, a driver monitoring system was also implemented as another source of data. Waste collection truck drivers were asked to fill in a manual form every time they passed by the monitored bins. Note that when the driver is performing the collection route of a certain recyclable material (collection routes are mono-material as the trucks have only one compartment), he/she also records the fill level of bins of other recyclable materials when bins are grouped together in the same location as is very often the case. The manual form had fields for the driver’s name, date, time, bin ID, fill level and occurrence of collection, as shown in Figure A-4 (Supplemental Appendix). Each row of the manual form corresponds to a unique observation. Additionally, to aid the drivers in their monitoring task, the inside of each bin was marked at the levels corresponding to integer multiples of 25%. The 25% levels were chosen because the drivers were already accustomed to them (as part of their jobs, they were already asked to record bin fill levels along collection routes, but this information was not being used by the company). This allowed for easier, faster and more consistent visualization of the fill level.

### Data processing: Statistical analysis

A statistical analysis was performed to compare the quality of the data obtained from the sensor observations and the VO. We aim to quantify the correlation between the time series data collected by the drivers and the sensors (VO vs sensor observations, respectively). In particular, the time series analysed in this study are irregular because they are sampled asynchronously and at irregular intervals. An irregularly spaced time series is a sequence of observation time and value pairs $\left(t_{i},x_{i}\right)$ of length N , where $x_{i}$ is the value at time $t_{i}$ with $t_{1}<t_{2}<\ldots <t_{N}$ , and the difference between consecutive observation times is not constant (Rehfeld et al., 2011). As mentioned before, the sensors report at least one observation per day. Conversely, the truck drivers would record observations when they performed a route which included the monitored bins. This generally occurred with intervals of 3–5 days. Additionally, the VO were made between midnight and 03.00. Given these conditions, VO rarely coincided with sensor observations. Therefore, sophisticated techniques were required to estimate the correlation coefficient for the time series.

A comparison of different correlation analysis methods for irregularly sampled time series is presented by Rehfeld et al. (2011). The first method consists of resampling both time series onto a common regular time grid with constant time interval. The authors use the time grid

(1) $$\begin{array}{c} \Delta t=\max\left(\bar{\Delta t^{x}},\bar{\Delta t^{y}}\right) \end{array},$$

where $\bar{\Delta t^{x}}$ is the mean sampling interval for time series x and $\bar{\Delta t^{y}}$ is the mean sampling interval for time series y . Once the regular time grid is determined, a linear interpolation is calculated for the observed values in each time series, after which standard correlation analysis methods can be applied. Another method studied by the authors is correlation slotting. This method consists of sorting the observations into slots of width Δτ . First, the observations for each time series must be standardized so that they have zero mean and unit variance. Then, only slots which contain observations from both time series are considered by the correlation estimator. The authors used equation (1) for the slot width, to increase the probability that at least one observation from each time series exists in the slot. Finally, the cross-correlation of the slotted standardized observations is calculated. In addition, the authors considered non-rectangular kernels, namely the sinc kernel and the Gaussian kernel. Each of these kernels weigh the observations by their distance to the centre of the slot, instead of strictly considering observations contained in each slot and assigning them equal weights. The tests performed by the authors indicate that the Gaussian kernel has the best performance. In this study, we opted by using the resampling approach due to its simple implementation and good performance reported by Rehfeld et al. (2011). For the resampling method, the linear interpolation technique was used for the reasons discussed by Rehfeld et al. (2011), that is, the effects of other standard routines are not much different in their variance reduction towards the high-frequency end of the spectrum (Schulz and Stattegger, 1997). Finally, the correlation between the two resampled time series was estimated using Pearson’s correlation coefficient (2) (Rodgers and Nicewander, 1988)

(2) $$\begin{array}{c} r_{xy}=\frac{\sum_{i=1}^{n}(x_{i}-\bar{x})(y_{i}-\bar{y})}{\sqrt{\sum_{i=1}^{n}{\left(x_{i}-\bar{x}\right)}^{2}\;\sqrt{\sum_{i=1}^{n}{\left(y_{i}-\bar{y}\right)}^{2}}}} \end{array}$$

Where n is sample size, $x_{i},y_{i}$ are the individual sample points and $\bar{x}$ , $\bar{y}$ the sample mean for both time series x and y .

### Proposal: Mobile Sensors (MS)

Given the pros and cons of SS and VO (see Table 1), we propose a third one: a hybrid approach involving the use of MS to accurately measure fill levels (removing human error factors) coupled with an adaptive predictive model (to better estimate future fill levels, as observations are only made when the bin is visited). The concept of this approach is to have a mobile fill level sensor in each waste collection truck with which the truck drivers would measure the bin fill levels. Each bin would have a dedicated opening or attachment point for the MS, to make consistent fill level observations. Altogether, this hybrid approach can offer considerable gains for waste collection companies, reducing the number of sensors required as well as reducing their maintenance cost—a MS can have a rechargeable battery and is not exposed to the elements such as a bin-SS. Furthermore, it balances the advantages and disadvantages of the other two approaches, giving the accuracy and precision of sensor observations at a fraction of the cost, but with a reduced observation frequency, thus requiring an adaptive predictive model.

### Modelling: Prediction model and trade-off analysis

The VO and the MS need a short-term predictive model to assess how the monitoring approaches will operate in terms of number of collections and overflows, as they do not rely on real-time information (unlike the SS). For this, we chose a Gaussian process (GP) as the predictive model to use, as in the work of Ferrer and Alba (2019) three models were used to predict daily bin fill levels (linear regression, GP and support vector machine for regression) and the authors found that the GP model was the best predictor. We highlight that the predictive model is not a contribution of this work, but it is a necessary tool to perform the trade-off analysis. Therefore, we follow the work of Ferrer and Alba (2019) in this matter.

A GP is specified by a mean function and a covariance function. In the work of Ferrer and Alba (2019), the authors do not specify which covariance function (also known as kernel) was used for the GP. Therefore, a suitable kernel was designed for this study based on the in-depth analysis presented by Rasmussen and Williams (2006).

**Figure 2.**
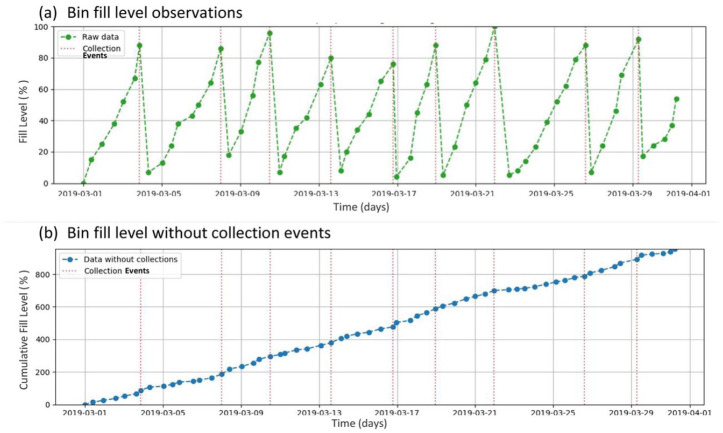
Pre-processing step applied to the visual and sensor observations before the GP regression is calculated: (a) bin fill level observations and (b) bin fill level without collection events.GP: Gaussian process.

In order to understand the choice of kernel, we must first describe the pre-processing step applied to both the VO and the sensor observations. This step consists in removing the occurrence of waste collections from the time series. It is necessary to perform this operation because the intervals between collection occurrences are irregular (in our study, intervals between collections ranged from 2 days to ten days), and the predictive model must work independently of them. Additionally, the GP kernel can be simplified for the data with collections removed, as it no longer requires a periodic component. Figure 2 illustrates this step with simulated data. Figure 2(a) shows a time series (in green) which could correspond to real bin fill level observations for the month of March 2019 (dates are given on the bottom axis). The dashed vertical red lines represent simulated collection events, leading to a large reduction in the bin fill level (although not necessarily to 0%, as this is often observed in the real data). In Figure 2(b), we see the bin fill level behaviour (in blue) if no collections had occurred, that is, the bin fills continuously. Figure 2(b) shows the cumulative fill level in percentage, meaning that, if in the period observed it reaches a value of 900%, the bin filled over nine times (900/100% = 9).

The collections are ‘removed’ by traversing the time series from the end to the start and, upon reaching a time t where a collection occurred, adding the fill level observed immediately before t to all the observations made after t . This operation is done in reverse order (latest date to earliest date) so that previous fill levels are not added multiple times.

Based on the arguments presented in the previous paragraphs, the predictive model used in this work is a GP with a kernel given by equation (3). The first term, $c_{0}(\sigma_{0}^{2}+x\cdot x\prime ),$ is a dot product kernel, parameterized by a hyperparameter $\sigma_{0}$ and scaled by a factor $c_{0}$ . The second term, $c_{1}{\left(1+\frac{x-x\prime^{2}}{2\alpha l^{2}}\right)}^{-\alpha }$ , is a rational quadratic kernel, parameterized by a length-scale parameter l and a scale mixture parameter α and scaled by a factor $c_{1}$ . The dot product term is used to model the long-term rising trend observed in the data (see Figure 2 (b)), whereas the rational quadratic term models the short-term changes (e.g. a waste deposition or compaction event). Additionally, the uncertainty in the observations is specified and accounted for in the GP regression by adding it to the diagonal of the kernel matrix (Rasmussen and Williams, 2006).

(3) $$\begin{array}{c} k(x,x\prime )=c_{0}(\sigma_{0}^{2}+x\cdot x\prime )+c_{1}{\left(1+\frac{x-x\prime^{2}}{2\alpha l^{2}}\right)}^{-\alpha } \end{array}$$

The predictive model was implemented with scikit-learn (Pedregosa et al., 2011), which uses the gradient ascent method to optimize the kernel hyperparameters. Recognizing this, the initial value of all the hyperparameters is set equal to 1.0 with lower and upper bounds of 1.0 × 10^−10^ and 1.0 × 10^5^, respectively, except for α that was set with an initial value of 0.125.

For each time series, the GP was trained on the initial 75% of observations (training set), and then it was used to make predictions in the time period corresponding to the remaining 25% of observations (test set). This predictive model was used in both the VO and the MS monitoring approaches. It was not used in the SS monitoring approach because, in that approach, it is assumed that the fill level reported by the sensors is used to determine whether the bin should be collected or not (thus, no prediction is required). In the case of the MS monitoring approach, the SS observations were subsampled to achieve an observation frequency similar to that of the VO, and they were linearly interpolated due to the fact that the SS observation times rarely coincided with the VO times.

The comparison of the performance of each monitoring approach can be made with a trade-off analysis based on the collected data. We can demonstrate that for different values of a parameter which encodes how conservative we want to be with respect to the risk of bin overflowing, a trade-off can be made in terms of the number of overflows versus the number of collections. Naturally, a reduction in one of these numbers generally leads to an increase in the other. Moreover, a reduction in the number of collections implies a reduction in the length or number of collection routes, which results in a reduction in the cost of operations. However, it also increases the risk of bins overflowing, which impacts the company’s service level, and can expose it to serious fines (Mes et al., 2014).

To estimate this trade-off based on real data, several steps were taken. First, for the VO and the MS monitoring approaches, it was necessary to train the predictive model. Given the different number of observations (see Table 2) and the irregular observation times for each time series, the training/test periods for each bin and monitoring approach were slightly different. Nonetheless, the test period generally started at the beginning of August 2019. In order to have comparable results, the test period for the SS monitoring approach was set to begin on 1 August 2019.

**Table 2.** Results of the data collection phase.

		Glass	Plastic	Paper 1	Paper 2
Sensor observations	Total	91	690	695	372
Visual observations	Total	124	169	179	156
	Outliers	13	19	16	25
	Utilized	111	150	163	131
Collections	Real	11	66	67	39
	Min./0 overflows (sensor)	N/A	44	42	15
	Min./0 overflows (visual)	3	39	42	11
Overflows	Real	0	20	20	2

N/A: Not Applicable.

Furthermore, to be able to compare the monitoring approaches with the current situation (current situation is designated by what actually happened in the observed period in terms of the number of collections made by the company and the number of overflows reported), collections are simulated within the test period based on a mathematical rule defined for each monitoring approach. From a survey of the real collection times, it was found that collections have generally occurred between 01.00 a.m. and 02.00 a.m. As such, simulated collections were set to always occur at 02.00 a.m. for consistency and to approximate the real collection behaviour.

For the SS approach, the rule to determine when a collection should occur was to check the fill level each day at 5.30 p.m.; if it is greater than the threshold value $f_{th}$ perform a collection on the next day at 02.00 a.m., otherwise do nothing. As we did not have sensor observations every day at 5.30 p.m., simulated sensor observations were generated by linear interpolation between the two closest real sensor observations (e.g. if we have a sensor observation at 4.00 p.m. reporting a fill level of 40%, and the next observation at 7.00 p.m. reporting a fill level of 50%, the linear interpolation will provide an estimate of the fill level at 5.30 p.m. (fill level 45%). Note that we do not intend to extrapolate the fill level between these two observations using a very detailed model, but simply estimate a plausible value between two observations to model how the SS approach could be applied in practice. The same linear interpolation procedure was used when collections occurred, to determine whether there was an overflow and to calculate the subsequent fill levels, as simulated collections rarely coincided with real collections.

For the VO approach, the predictive model was used to determine when the next collection should occur. The rule we defined to determine when a collection should occur was to calculate the GP prediction each day at 02.00 a.m. and the probability that an overflow has occurred (i.e. the probability that the fill level is greater than 100%); then, perform a collection on the first day where the probability is greater than the specified threshold probability $p_{th}$ . As the VO did not match the model observation times exactly, simulated observations were estimated by linear interpolation between the two closest real observations and rounding off up to the nearest multiple of 25% (to simulate the real VO).

For the MS approach, the predictive model was also used to determine when the next collection should occur, and the rule to determine when a collection should occur was the same as that described for the VO approach. To simulate reasonable sensor observations in this approach, the SS observations were subsampled to have a similar sampling frequency to the VO. As the SS observations did not match the VO time exactly, simulated observations were estimated by linear interpolation between the two closest SS readings and rounded to the nearest percentage point.

## Study results

### Data collection

Two drivers collected the data considered in this study. Driver A performed the collection routes for Paper, and Driver B performed the collection routes for Plastic. Although each driver collects a specific material, both drivers recorded the fill levels for all three materials. Driver C, who performed the collection route for glass, was not involved in this experiment as very few observations would be recorded by him (glass is only collected once a month on average). An initial ‘learning period’ was considered for the VO, where several irregular observations were noticed, so the first 2 weeks of observations were discarded from all analyses. As such, the analysed interval was 1 March 2019 to 30 September 2019. The number of sensor and VO, as well as the number of collections and overflows for each bin, is shown in Table 2. Notice that the number of sensor observations is almost three times greater than the number of VO. The collections and overflows were reported by the drivers in the manual forms.

It should be noted that the glass bin has fewer sensor observations for two reasons: first, its fill rate is comparatively small and close to linear, meaning that it would fill up slowly and consistently over time; secondly, there was a sensor malfunction on 16 June 2019 after which the sensor was no longer operational.

Additionally, based on the time series data and the method illustrated in Figure 2, it was possible to estimate the minimum number of collections required in order to have zero overflows. This can be considered an ideal situation, where a collection occurs as soon as the bin is 100% full. These values are also shown in Table 2. The N/A mention given for the glass bin is due to the sensor malfunction.

It can be observed in Table 2 that, over the period analysed, 20 overflows have occurred for both the plastic and paper 1 bins, representing approximately 30% of the number of collections for each bin. Moreover, the minimum possible number of collections required for zero overflows to occur is approximately equal to two thirds of the number of collections (67% for the plastic bin and 63% for the paper 1 bin). This means that more collections were performed than what could be considered ideal, yet overflows still occurred. This is due to the static routes used by the waste collection company, which do not take place at the right time, that is, at times when bins are not close to being full or when they have already overflowed. Another point to note in Table 2 is the small number of sensor observations for the paper 2 bin. This bin had a slower fill rate than the paper 1 bin, probably due to their different locations, thus resulting in fewer sensor observations (as the sensors do not report small changes in fill level) as well as fewer overflows – only two for the period analysed.

### Comparative statistical analysis

In order to demonstrate the value of different types of observations (sensor and visual), a comparative statistical analysis was performed. The correlation coefficients and its statistical significance (*p*-values) are shown in Table 3. In addition to the correlation between the total VO dataset and the sensor dataset (shown in the first row), the correlation between the VO of each driver and the sensor observations was calculated.

**Table 3.** Correlation between the visual observations and the sensor observations using the method developed by Rehfeld et al. (2011).

	Glass	Plastic	Paper 1	Paper 2
All visual observations				
Correlation	0.26	0.57	0.49	0.53
*p*-Value	1.67E-06	6.02E-55	1.14E-38	4.77E-47
Driver A’s observations				
Correlation	0.36	0.09	0.48	0.50
*p*-Value	6.44E-11	0.02	1.91E-37	1.63E-39
Driver B’s observations				
Correlation	0.24	0.61	0.13	0.23
*p*-Value	8.60E-06	7.70E-63	0.00	7.64E-09

The Pearson correlation coefficients between the sensor observations and the VO, given in Table 3, raise two points. First, a moderate degree of correlation can be found between both types of observations (values around 0.5 are observed, and all *p*-values are less than 0.05; meaning that for a significance level of 0.05, there is sufficient evidence to conclude that the correlation coefficients are significantly different from zero), considering that the VO were made in intervals of 25%, which represents a low precision. This indicates that, assuming that the sensor observations have a high accuracy, VO still convey a considerable amount of information. Second, if we look at the bottom two rows of Table 3, we see that driver A, who was responsible for collecting the paper 1 and 2 bins, made observations which are more highly correlated with the sensor observations for those bins (0.54 and 0.51 vs 0.14 and 0.24, respectively); likewise, driver B, who was responsible for collecting the plastic bin, made observations which are more highly correlated with the sensor observations made for that bin (0.56 vs 0.08). This suggests that the driver who is responsible for collecting a certain bin makes more accurate observations for that bin; however, as the sample size is small, we cannot draw a conclusion.

### Predictive model

**Figure 3.**
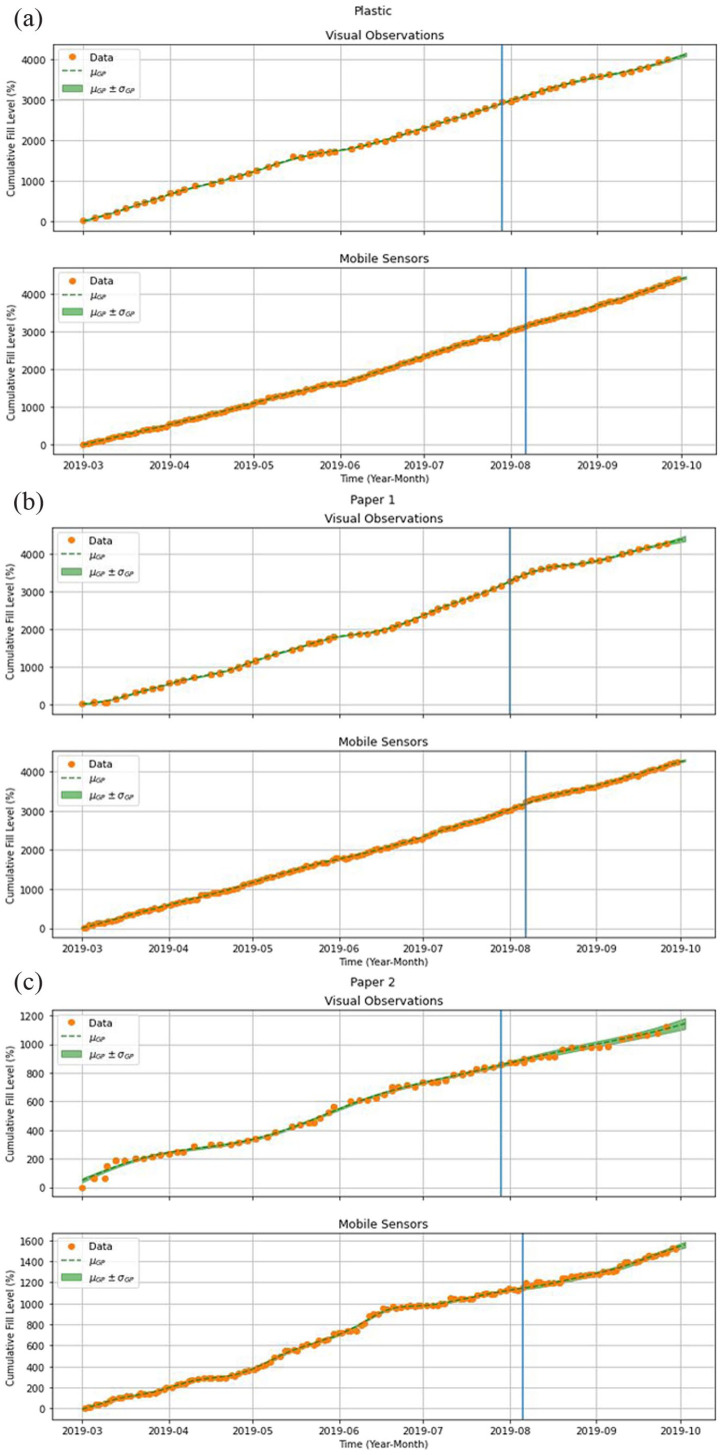
Final state of the VO and MS predictive models for the plastic, paper 1 and paper 2 bins, assuming that bins fill continuously – without collection events. The orange dots are the observations, and the green curves are the predictions. The vertical blue lines represent the separation between the training set and the test set. VO: visual observations; MS: mobile sensors.

The charts in Figure 3 show the final state of the predictive model for each bin (paper 1 and 2 and plastic), in both the VO and the MS monitoring approaches (green curves). Please note that, as explained in section ‘Proposal: MS’ and illustrated in Figure 2, the collection events are not considered; we are modelling the bin fill level behaviour if no collections occur. The glass bin was excluded from this analysis because of its slow fill rate (see Table 2: only three collections were required for the whole period between 1 March and 30 September 2019), as well as because of the discontinuation of sensor observations due to the sensor malfunction. The vertical blue lines in each chart represent the limit between the training set (containing the initial 75% of the time series) and the test set (containing the remaining 25%). As the observations for each time series were made at different times and in different quantities, those lines do not coincide. Furthermore, although it may seem that the MS charts contain more observations, this is simply due to the fact that the VO are usually made in pairs, and so they overlap each other. As noted in section ‘Study methodology’, the sensor observations were subsampled to have a mean observation frequency similar to that of the VO. It can be seen in Figure 3 for plastic and paper 1 that the predictive model trained with the VO datasets follows the curve of the data points and has more non-linear contributions (maybe because VO are reported in a discrete scale of multiples of 25%, see Figure A-4 in Supplemental Appendix); conversely, the predictive model trained with the MS datasets is essentially linear. However, for paper 2, we can see that the data points themselves are more irregular and, consequently, both predictive model curves show considerable non-linearity.

Table 4 shows two measures of prediction accuracy: mean absolute error (MAE) and mean absolute percentage error (MAPE). Low values for either MAE or MAPE can be observed, which validates the predictive model.

**Table 4.** Prediction accuracy measures.

	Plastic	Paper 1	Paper 2
Prediction w/visual observations			
MAE	0.152	0.115	0.105
MAPE	0.4%	0.3%	1.1%
Prediction w/sensors observations			
MAE	0.122	0.136	0.141
MAPE	0.3%	0.4%	1.1%

MAE: mean absolute error; MAPE: mean absolute percentage error.

### Trade-off analysis

**Figure 4.**
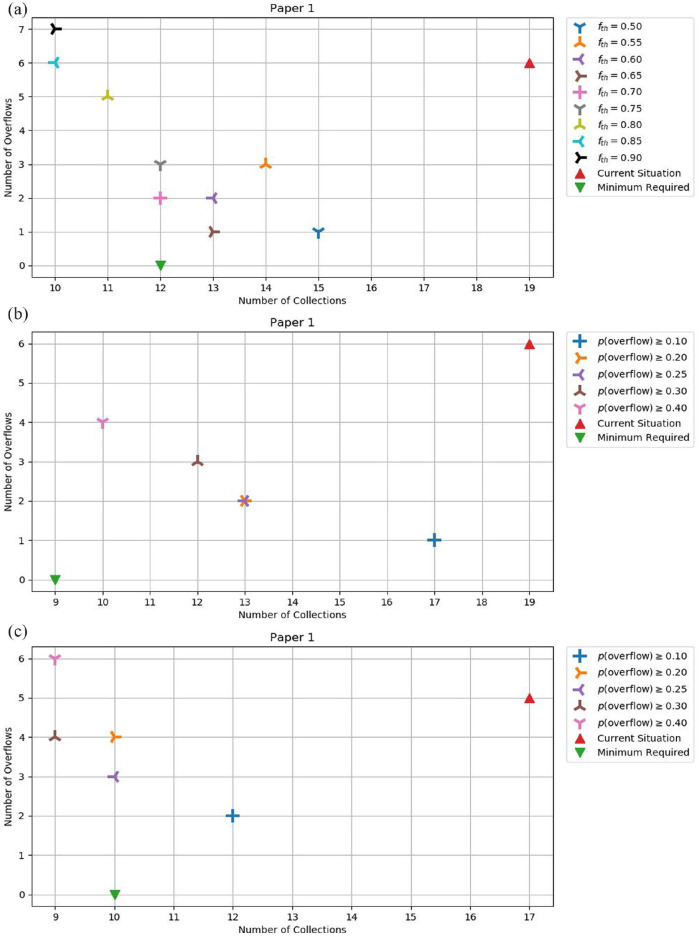
Trade-off between collections and overflows in the three monitoring approaches for the paper 1 bin: (a) static sensors, (b) visual observations and (c) mobile sensors.

The charts in Figure 4 illustrate the results obtained for the three monitoring approaches for the paper 1 bin, for different values of $f_{th}$ and threshold probabilities. For the remaining bins, please see Figures A-5 and A-6 (Supplemental Appendix). For each chart, the horizontal axis shows the number of simulated collections, and the vertical axis shows the number of overflows that would have occurred, based on the real data. Additionally, every chart has a red triangle labelled ‘Current Situation’ representing the number of real collections and overflows that occurred in the test period (1 August to 30 September), and a green triangle labelled ‘Minimum Required’, representing the minimum number of collections necessary to avoid overflows. The minimum required number is computed based on the method illustrated in Figure 2, where the collections are ‘removed’ from the time series data, and the bin fills continuously. If, for example, a bin reaches a value of 900% in the test period, it means that the minimum required collections in order to have zero overflows would be nine collections. This can be considered an ideal situation, where a collection occurs as soon as the bin is 100% full (as explained in section ‘Data collection’3.1).

#### SS monitoring approach

Recall that the SS monitoring approach assumes that there is a sensor installed in every bin and, therefore, we have a priori knowledge of the bin fill levels. As an example of how the collection rule works in the SS approach, assume that $f_{th}=0.60$ and that on day t , at 5.30 p.m., a bin has a fill level of 56%. As this is below the threshold level ( 0.56<0.60) , no collection occurs. If the fill level were 83%, a collection would be simulated on day t+1 at 02.00 a.m. Overflow occurrence is determined by the interpolated fill level at the time of collection (continuing the previous example, if at 02.00 a.m. on day t+1 the interpolated fill level was 105%, then the bin would be considered in overflow).

In the SS monitoring approach, there is a considerable investment to make as the cost of each individual sensor is high (around 100€–200€, according to Beigl et al., 2005; Vishnu et al., 2022), and locating them in the large number of bins that often characterizes waste collection systems implies a high investment (e.g. ERSUC owns around 15000 bins, representing an investment of more than 1.5 M€). However, this investment is counterbalanced by the availability of a priori information, which enables waste collection companies to deal with unexpected situations that the predictive model is unable to identify (e.g. a sudden increase in fill rates due to a local event). As such, this approach leads to a good performance in general. For example, in Figure 4(a), it can be observed that, for the threshold $f_{th}=0.65$ (brown symbol), the SS approach is as close as possible to the minimum required number of collections to have zero overflows (green triangle). This can be considered a balanced solution, which reduces the number of collections by 32% and reduces the number of overflows by 83%, compared to the current situation (red triangle). However, if we are willing to maintain the number of overflows equal to the current situation, and wish to minimize the number of collections as much as possible, we can use the threshold $f_{th}=0.85$ (light blue symbol), which leads to a reduction of 47% in the number of collections made.

#### VO monitoring approach

As mentioned above, the VO monitoring approach assumes that, each time a bin is collected, the driver observes and registers the fill level (before emptying the bin) and, therefore, we have a posteriori knowledge of the bin fill levels. As such, a predictive model is used to determine when the next collection should occur. As an example of how the collection rule works in the VO approach, assume that the threshold probability of overflow is 25% and that on day t the probability that a certain bin has overflowed is 15%. As this is below the threshold, no collection occurs. Next, assume that at 02.00 a.m. on day t+1 the GP predicts that the probability of overflow is 30%. As this surpasses the threshold probability of overflow, a collection is simulated on day t+1 at 02.00 a.m. Whenever a collection is simulated, a visual observation is also simulated. If the simulated fill level is greater than 100%, the bin is considered in overflow.

In the VO monitoring approach, the required investment is null as this is done by the truck drivers during the collection operation. However, the precision of the observations is small (multiples of 25%, compared to multiples of 1% for sensor observations) and the information is only known a posteriori. Nonetheless, this approach still provides a considerable improvement over the current situation and can be considered more conservative than the SS approach, as it prioritizes making more collections to avoid overflows. For example, in Figure 4(b) we can consider that, if we are willing to accept a certain number of overflows, the threshold $p_{th}=0.40$ (pink symbol) leads to the best solution in terms of number of collections, representing a reduction in collections of 47% and a reduction in overflows of 33%, compared to the current situation. If, however, we wish to reduce the risk of overflow, then the threshold value $p_{th}=0.10$ has the best result in the number of overflows, but with a reduction of only 11% (instead of 47%) in the number of collections.

#### MS monitoring approach

The MS monitoring approach assumes that each time a bin is collected, the driver uses a sensor to measure the fill level and, therefore, we have a posteriori knowledge of the bin fill levels. The measured value is more accurate than in the VO approach. Again, a predictive model is used determine when the next collection should occur.

In the MS monitoring approach, the investment is moderate, although it is considerably smaller than in the SS approach as the number of sensors to use in the trucks is much smaller (e.g. ERSUC owns a vehicle fleet of around 50 trucks, meaning that only 50 sensors would be needed, so the investment would be reduced to 5 k€). The precision of observations is the same as in the SS approach, but the information is known a posteriori and with the same observation frequency as the VO. The results for this approach show that it performs worse than the other two approaches, presenting larger numbers of overflows and collections. This is most likely due to a high confidence in the predictive model, which leads to a riskier behaviour, that is, fewer collections, resulting in a greater number of overflows. Nevertheless, Figure 4(c) still shows that the MS approach is an improvement over the current situation. In general, the number of overflows is comparable to the current situation, but the number of collections is greatly reduced.

#### Final remarks on the trade-off analysis

The analysis of the charts of Figure 4 and Figures A-5 and A-6 (Supplemental Appendix) results in a clear conclusion: either one of the monitoring approaches under study leads to significant improvements over the current situation, reducing both the number of collections and the number of overflows. The trade-off between the number of collections and the number of overflows is dependent on the respective threshold parameter, which can be tuned, allowing companies to choose how conservative or risky they want to be, depending on the cost of overflows and collections.

**Figure 5.**
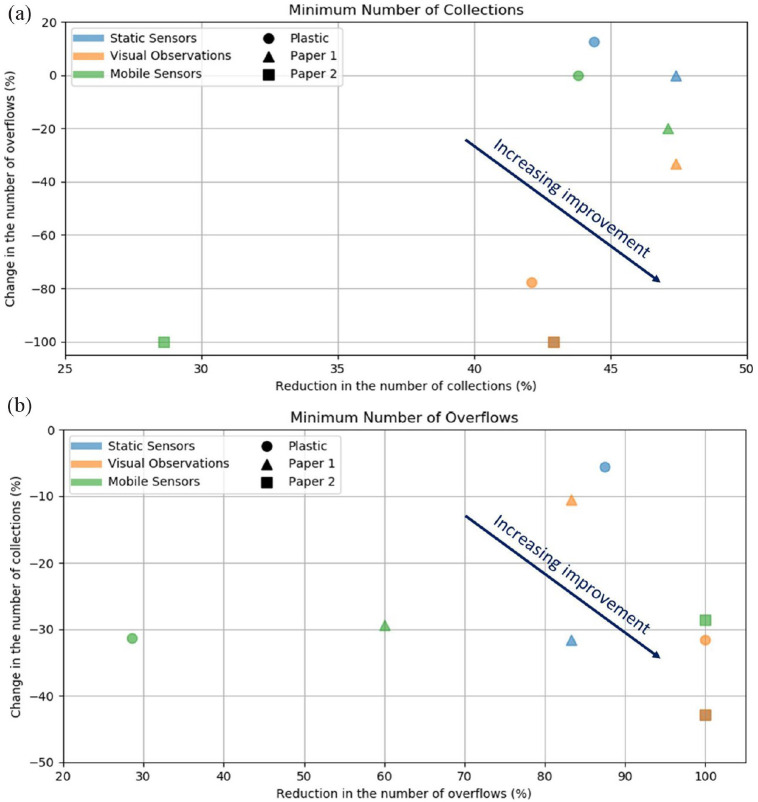
Comparison of the three monitoring approaches in terms of the (a) minimum number of collections achieved, and (b) minimum number of overflows achieved.

The charts in Figure 5 compare the gains achieved by the three monitoring approaches. Figure 5(a) illustrates the situation where the collections are minimized, that is, as different threshold values were used, different results were obtained. In Figure 5(a), we present the results provided by the threshold value that resulted in the minimum number of collections. It can be seen that the VO approach leads to the greatest simultaneous reduction in collections and overflows for paper 1 (orange triangle). For paper 2, both VO and SS gave the same results (brown square, as orange overlap blue square). For plastic, the VO approach provided the greatest reduction in the number of overflows (−79%) and also a large reduction in the number of collections (−42%) (orange circle). Figure 5(b) illustrates the situation where the overflows are minimized. Again, we present the results provided by the threshold value that resulted in the minimum number of overflows. Comparing the three approaches, the VO provided better results for plastic, SS provided better results for paper 1, and both (VO and SS) provided the same best result for paper 2. Therefore, to minimize the number of overflows, SS and VO approaches are on par with each other. The MS approach presents the worst results in this situation, mainly due to the small reductions in the number of overflows, accompanied by moderate reductions in the number of collections.

### Discussion

Prior studies on waste bin monitoring solutions focused mainly on sensor’s systems, namely on the characteristics of the technological devices included in such systems and how accurate they are. There is a rich body of research on bin sensor systems, acknowledged by two recent surveys (Sosunova and Porras, 2022; Vishnu et al., 2022). To the best of the authors’ knowledge, there is no prior study addressing a comparison between automatic monitoring through technology and human monitoring. Thus, the findings regarding the comparative analysis of these two approaches, that are the focus of our study, cannot be compared with other studies.

The sensors used in our experiment are ultrasonic sensors that measure waste volume. Using this type of sensor is aligned with the study of Rovetta et al. (2009) which concluded that measuring waste volume is what impacts the most the collection operation’s decisions. The authors presented a complete sensor and server system based on a set of electronic components installed in each bin, including a camera, an ultrasonic sensor, a temperature sensor, weight sensors, a processing unit and a telecommunications antenna. The studied system was shown to provide plenty of information about the bin state (fill level, temperature, humidity, waste density). However, the authors concluded that, although waste density is relevant for determining the number of bins to collect (as trucks have a maximum load capacity), it is the waste volume that determines whether a bin overflows or not, that is, the fill level ultimately determines which bins must be collected. Karadimas et al. (2016), Ramson and Moni (2017) and Yerraboina et al. (2018), also used ultrasonic sensors and wireless networks to sense and transmit fill-level data.

In our study, the accuracy of the sensors *per se* was not evaluated. Nonetheless, there are some studies that explore that subject. Mamun et al. (2016) tested the accuracy of ultrasonic sensors by placing mock waste bags in a bin and verified that it presented an error in the range of 5–10%.

Several approaches have recently been proposed in the literature to predict waste generation. There are approaches that work on long time scales (months) and apply to large areas (municipalities), notably the works of Abbasi and El Hanandeh (2016) and Kannangara et al. (2018), but only one that applies to individual bins on a daily basis (Ferrer and Alba, 2019) as in our study.

Ferrer and Alba (2019) propose a system to predict individual bin fill levels based on historic data, and to plan routes based on the predicted fill levels using an evolutionary algorithm. The data used in the tests comprised fill levels for 217 containers over an eleven-month period. These data were reported by the waste collection truck drivers for each collected bin. However, nothing is said about how truck drivers measured and registered the bin fill levels. The system was validated in a living laboratory scenario with the collaboration of a waste collection company. The authors concluded that the proposed system could reduce the length of collection routes by one-third compared to the static routes designed by the company, while avoiding visits to bins that were predicted to be below 50% of their capacity.

In our study, only four bins were monitored, so the impact in terms of collection routes (and route length) was not possible to access as in Ferrer and Alba (2019). Nonetheless, we report the reduction in terms of number of collections (up to 50%) what will impact the collection routes. Ferrer and Alba (2019) reported a MAPE of 3.83% for their predictions, and in our study, we reported 0.3–1.1% what represents even more accurate results. The reduction in the number of overflows is not reported in Ferrer and Alba, while our study reports an expected reduction of up to 100%.

## Conclusions

We presented a comparative study of three waste bin monitoring approaches based on data collected in a living laboratory scenario. The data comprised two sets of observations: sensor observations (provided by volumetric sensors installed in the waste bins); VO (provided by waste collection truck drivers). The study is exploratory because it is based on a limited number of bins as well as of drivers. Hence, its conclusions are preliminary.

As a first step to verify the utility of both types of observations, a comparative statistical analysis was made using a linear correlation estimation method for irregular time series. This analysis revealed a moderate degree of correlation, considering the low precision of the VO (each observation corresponded to a 25% interval). This analysis also identified that the observations made by the driver who was responsible for collecting a specific bin had a much larger correlation with the sensor observations than the observations made by the other driver. Thus, the visual observation process can be simplified by asking each driver only to register the fill levels of the bins they are responsible for collecting (as opposed to registering the fill levels for all bins found along their route). It is likely that incentives for good VO would increase their accuracy.

Upon simulating three monitoring approaches (SS, VO and MS), a trade-off analysis was made in terms of number of collections versus the number of overflows. A preliminary conclusion to draw from this analysis is that the implementation of any one of the approaches would lead to improvements over the current situation – that is, decreases in the order of 50% in both the number of collections and the number of overflows for the bins analysed. Furthermore, by varying a parameter which encoded risk aversion towards overflows, a trade-off could be seen where an increase in the number of collections was accompanied by a decrease in the number of overflows, and vice-versa. This trade-off allows waste collection companies to adapt their collection operations depending on the relevant costs: if the cost of overflow is low, then they can perform fewer collections with a higher risk of overflows; alternatively, if the cost of overflow is high, they should perform more collections to decrease the risk of overflows. From this preliminary comparison of the three approaches for the bins analysed, it is found that the best results are obtained for the VO monitoring approach. This is a rather unexpected result that could be due to a bias in the VO made by the drivers. Additionally, it could be due to the different rules used to determine when simulated collections should occur. Using the predictive model in the SS monitoring approach could improve its results; this analysis is left for future work.

Although this work has important preliminary conclusions, it has some limitations. The main one was already mentioned: all the data was collected by two drivers from four bins, representing a small sample. Moreover, the data utilized in the trade-off analysis came from only three of the four bins, and the bins were in the same street, which may have introduced a bias. Finally, due to the small number of bins monitored, vehicle routes were not considered. Routing considerations may change the trade-offs. Nonetheless, this study can be seen as a first exploratory test that should be further extended at a wider scale where a full region (e.g. an entire municipality, as typically the collection routes are defined by the planners considering municipal boundaries) could be monitored both by sensors and by drivers to retrieve more general results.

Therefore, future work is needed to assess the monitoring approaches considered, and a larger number of bins should be monitored (and, consequently, with more drivers involved). With a larger number of bins over a large geographic area, routing problems can be considered, enriching the trade-off analysis. With more drivers involved, the ‘responsible driver bias’ founded in this exploratory study could be further investigated. Also, the optimization of the placement of a limited number of waste bin sensors could be explored. This is a necessary step towards smart cities considering that a completely sensorized network is still far in the future. There is very little work being done towards identifying the optimal placement of sensors. If a company has a network of 10,000 bins, and decides to install 500 sensors, it is important to know which bins should be sensorized to minimize uncertainty or provide the most relevant information. Such a problem can be combined with the work here developed, namely, by installing sensors in some bins and monitoring other bins using the VO or the MS monitoring approaches.

## Supplemental Material

sj-docx-1-wmr-10.1177_0734242X231160691 – Supplemental material for Comparison of different waste bin monitoring approaches: An exploratory study Click here for additional data file. Supplemental material, sj-docx-1-wmr-10.1177_0734242X231160691 for Comparison of different waste bin monitoring approaches: An exploratory study by Yoeri Brouwer, Ana Paula Barbosa-Póvoa, António Pais Antunes and Tânia Rodrigues Pereira Ramos in Waste Management & Research
