# Supplementary material for: Comparison of different waste bin monitoring approaches: An exploratory study
Source: Waste Manag Res. 2023 May 3;41(10):1570–83. doi: 10.1177/0734242X231160691 (PMC10517583; doi:10.1177/0734242X231160691)
Supplement: sj-docx-1-wmr-10.1177_0734242X231160691 – Supplemental material for Comparison of different waste bin monitoring approaches: An exploratory study [file sj-docx-1-wmr-10.1177_0734242X231160691.docx]

**Appendix**


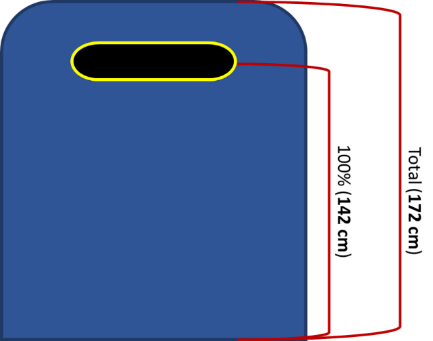


**Fig. A-1.** Total bin height and 100% fill level height.

**Fig. A-2.**: Waste collected per month (in ton) in the whole ERSUC area during year 2019 (source: company ERSUC)

| 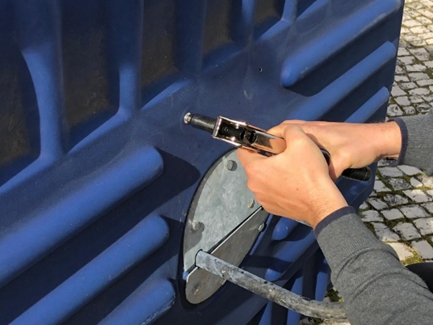 | 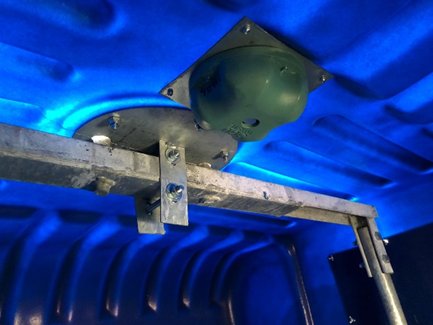 |
| --- | --- |

**Fig. A-3.** Installing a sensor in one of the bins with rivets.

| 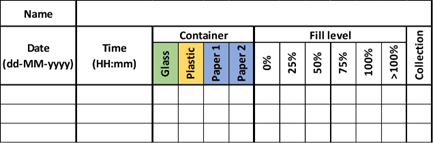 |
| --- |
| 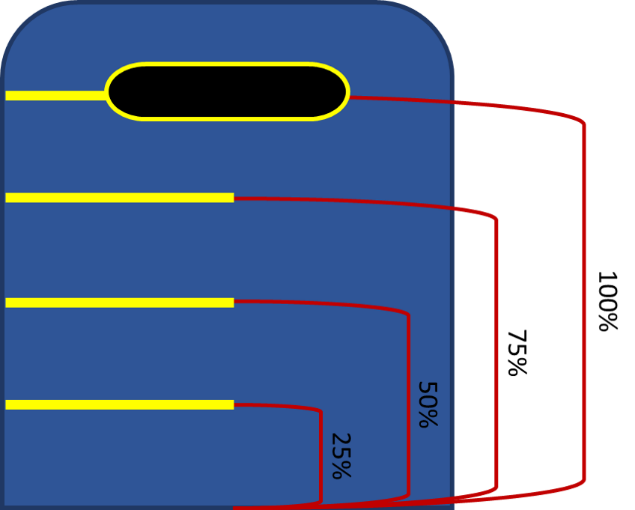 |

**Fig. A-4.** Manual form for fill level monitoring and schematic representation of the markings made inside each of the bins to aid the visual monitoring

| 1. Static Sensors   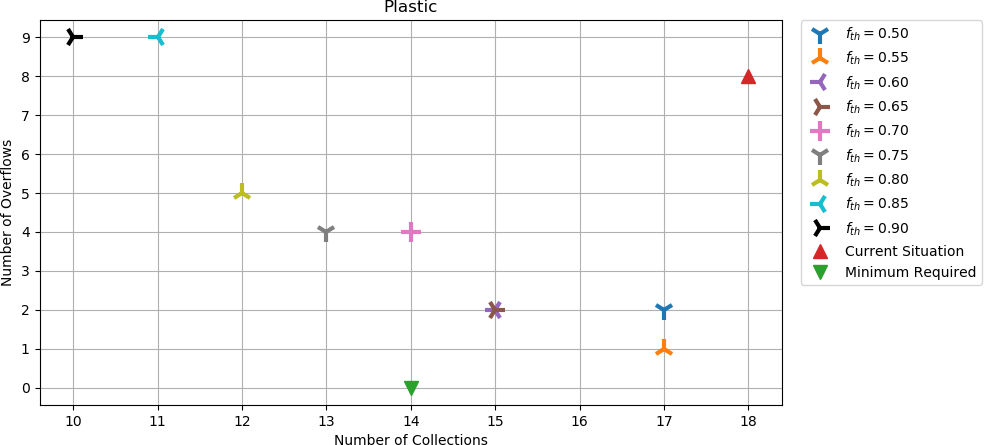 |
| --- |
| 1. Visual Observations   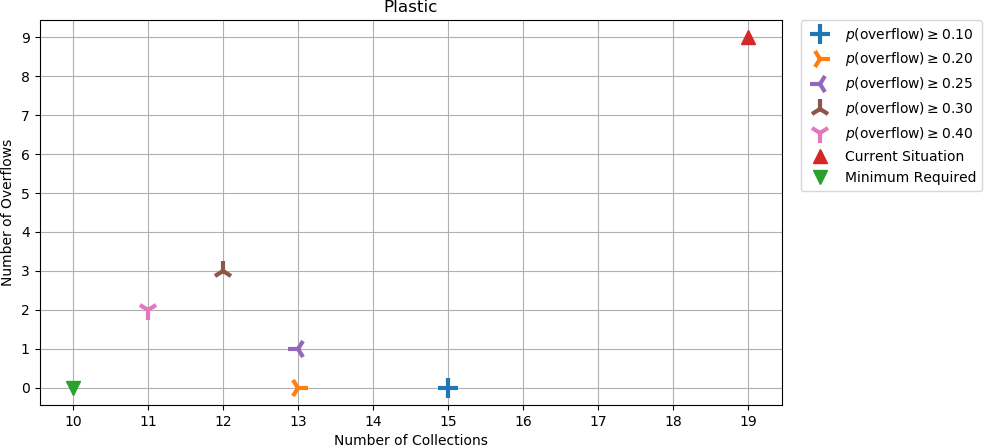 |
| c) Mobile Sensors  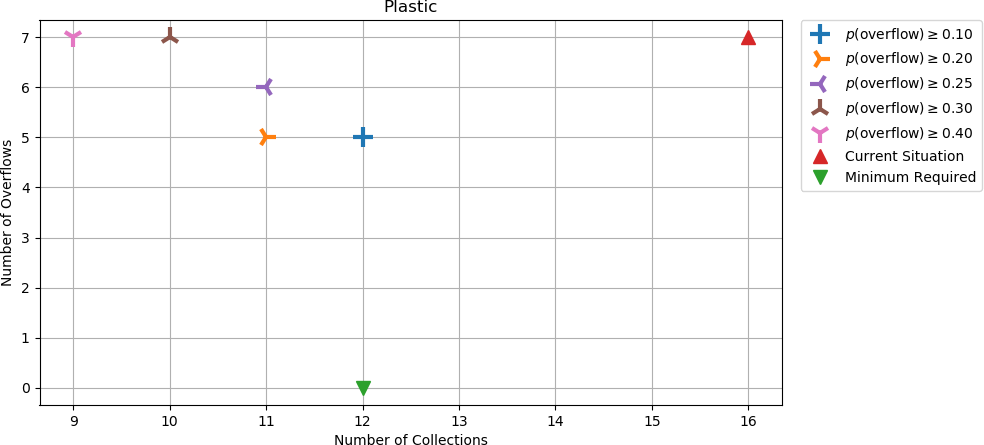 |

**Fig. A-5.** Trade-off between collections and overflows in the three monitoring approaches for the Plastic bin.

| a) Static Sensors  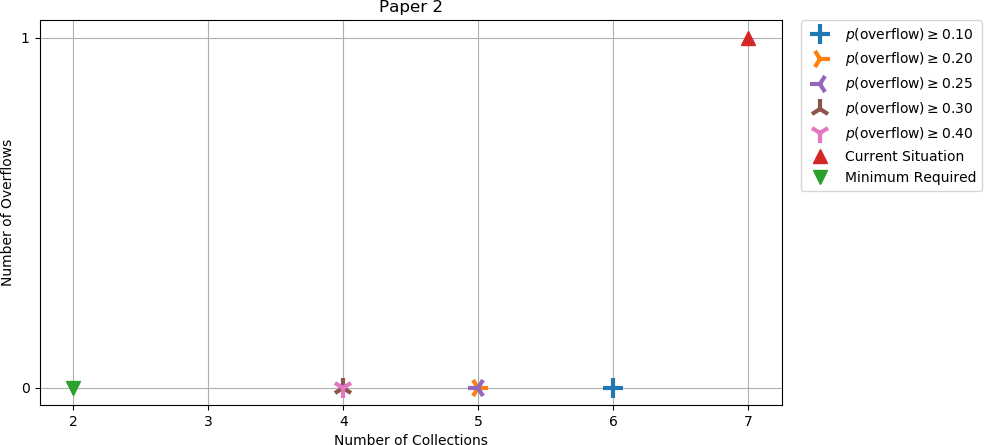 |
| --- |
| b) Visual Observations  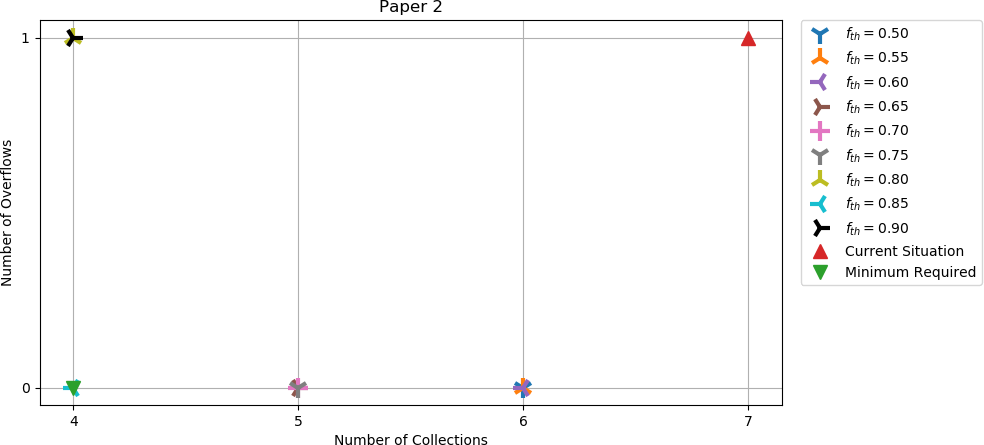 |
| c) Mobile Sensors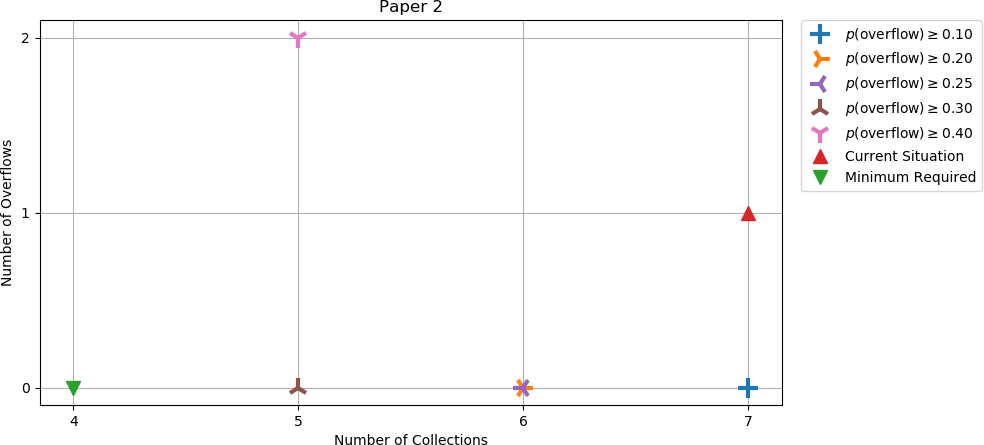 |

**Fig. A-6.** Trade-off between collections and overflows in the three monitoring approaches for the Paper 2 bin.
